# Positive and Negative Affect Mediate the Relationship Between Memory Lapses and Life Satisfaction

**DOI:** 10.1093/geroni/igab046.2202

**Published:** 2021-12-17

**Authors:** Jennifer Turner, Jacqueline Mogle, Nikki Hill, Sakshi Bharhava, Laura Rabin

**Affiliations:** 1 Pennsylvania State University, State College, Pennsylvania, United States; 2 Penn State University, University Park, Pennsylvania, United States; 3 Pennsylvania State University, University Park, Pennsylvania, United States; 4 Brooklyn College, The City University of New York, Brooklyn, New York, United States

## Abstract

Aging is associated with declines and challenges, yet better subjective well-being. Life satisfaction is one aspect of well-being that may be sensitive to daily challenges. Daily memory lapses (e.g., forgetting words or meetings) are common and relevant for many adults. How individuals emotionally respond to challenges like memory lapses is a factor that could determine whether these experiences affect well-being. In a coordinated analysis of two datasets (N=561; ages 25-93 years) using multilevel modeling, we examined whether affective changes related to memory lapses mediated the relationship between memory lapses and life satisfaction. Results were similar across datasets: memory lapses were associated with reduced positive affect and increased negative affect. These associated affective changes also mediated the relationship between lapses and life satisfaction. We discuss the potential implications of our findings for linking proximal events and distal outcomes, and potentially intervening and identifying common challenges to mitigate broad reductions in well-being.

